# Fluoroscopic evaluation of the influence of needle gauge on epidural spread in caudal block

**DOI:** 10.1097/MD.0000000000015896

**Published:** 2019-05-31

**Authors:** Woo Seog Sim, Hue Jung Park, Ji Hye Kwon, Min Seok Oh, Hyun Joo Jung, Min Kyoung Cho, Jin Young Lee

**Affiliations:** a Department of Anesthesiology and Pain Medicine, Samsung Medical Center, Sungkyunkwan University, School of Medicine; b Department of Anesthesiology and Pain Medicine, Seoul St. Mary's Hospital, The Catholic University of Korea, College of Medicine, Seoul, Republic of Korea.

**Keywords:** analgesic efficacy, caudal block, contrast, epidural spread, gauge, lower back pain

## Abstract

Caudal block has limited injectate distribution to the desired lumbar level due to the relatively long distance from the injection site and reduction in the volume of injectate due to leakage into the sacral foramen. The objective of this study was to investigate the influence of needle gauge on fluoroscopic epidural spread and to assess the correlation between the spread level and analgesic efficacy in patients undergoing caudal block. We retrospectively analyzed data from 80 patients who received caudal block for lower back and radicular pain. We categorized patients based on the epidural needle gauge used into group A (23 gauge), group B (20 gauge), and group C (17 gauge). Fluoroscopic image of the final level of contrast injected through the caudal needle and pain scores before the block and 30 minutes after the block recorded using a numerical rating scale, were evaluated. Of the 80 patients assessed for eligibility, 7 were excluded. Thus, a total of 73 patients were finally analyzed. Age, sex, body mass index, diagnosis, lesion level, lesion severity, and duration of pain did not differ among the 3 groups. All patients showed cephalic spread of contrast. Contrast spread beyond L5 was seen in 26.9% of patients in group A, 41.7% in group B, 39.1% in group C, and 35.6% overall; there was no significant difference among the groups (*P* = .517). Analgesic efficacy was not significantly different among the groups (*P* = .336). The needle gauge did not influence the level of epidural spread or analgesic efficacy in caudal block.

## 1 Introduction

Lumbosacral radicular pain is characterized by a radiating pain in the lumbar or sacral dermatomes.^[1]^ It is caused by irritation or compression of the affected nerve root. Caudal block is commonly chosen for the management of lower back and radicular pain due to the simplicity of the technique and relatively low risk of complications.^[2]^ However, despite its apparent safety, caudal block has the disadvantage of limited injectate distribution to the desired lumbar level due to the relatively long distance from the sacral hiatus and reduction in the volume of injectate due to some leakage into the sacral foramen.^[3,4]^ Moreover, lumbar pathology can result in resistance to injectate spread in the epidural space.^[5]^ Epidural fibrosis and adhesions are considered to interrupt nerve root nutrition and blood supply, which can prevent contact of the injectate with the affected nerve root.^[6]^ Hogan reported that the distribution and flow of solution in the epidural space is affected by increasing age, anatomical deformation, or compression resulting from degenerative disease, joint disease, or spinal stenosis.^[7]^ Caudal contrast flow pattern is variable and controversies still exist with regard to correlation of the flow with the extent of the block.^[8]^ There is no consensus with regard to the effect of needle gauge used for caudal injection on the spread of injectate and analgesic efficacy. In clinical practice, various needle sizes (from 17 to 25 gauge) have been used for epidural injection.^[4,6,8–12]^ Thus, the objective of this study was to investigate the influence of 3 different needle gauges on epidural spread and to assess the correlation between the spread level and analgesic efficacy in patients undergoing caudal block.

## 2 Methods

### 2.1 Patients

We retrospectively reviewed the electronic medical records of 80 patients who underwent caudal block from January 2017 to April 2018 in a single center. The age of the patients ranged from 21 to 86 years. All patients had lower back and/or radicular pain. The inclusion criteria were as follows:

1. a primary diagnosis of lower back pain with or without radiating pain to the lower limbs,
2. cross-sectional imaging study (either computed tomography or magnetic resonance imaging) of the lumbosacral spine indicating diagnoses of spinal stenosis, herniated nucleus pulposus, and/or degenerative spinal disorder.

The exclusion criteria were prior lumbar or caudal epidural injection within 2 months, history of lumbosacral surgery, lumbosacral neuroplasty, or neoplastic diseases. The lesion level was chosen based on clinical manifestation, physical examination, and review of imaging studies. This study was approved by our departmental ethics committee (SMC 2018-05-019) and was registered with CRIS (Clinical Research Information Service of the Korea National Institute of Health, http://cris.nih.go.kr/cris/index.jsp, registration number: KCT0002957).

### 2.2 Interventions

**Figure 1:**
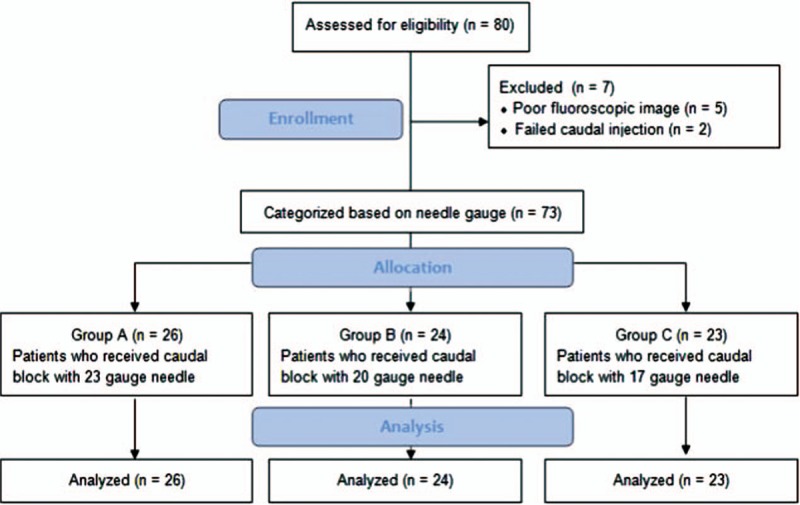
Flow diagram of the study.

All procedures were performed under fluoroscopic guidance by a single experienced pain physician. Patients were placed in the prone position with their feet rotated laterally and a pillow under the hip. Anteroposterior and lateral views were obtained with a C-arm (OEC series 9800, General Electric, USA) to ensure that the midline of the sacral hiatus was accurately determined. Following aseptic preparation and infiltration with 1% lidocaine, a 23, 20, or 17 gauge needle (Tae-Chang Industrial Co., Seoul, Korea) was inserted into the skin surface over the sacrococcygeal ligament midway between the sacral cornua. The 20 and 17 gauge needles were Tuohy needles and the 23 gauge needle was a spinal needle. With the needle bevel facing dorsally, the needle was angled at 60° to 90° to the skin surface, advanced until a ‘pop’ (piercing the sacrococcygeal ligament) was felt, lowered to a 20° to 30° angle to the skin, and then advanced an additional 2 to 3 mm into the sacral canal. After confirming negative aspiration, 3 ml of contrast medium (Omnipaque, 300mgI.ml^−1^, GE Healthcare, Little Chalfont, Buckinghamshire, UK) was instilled to confirm whether the needle was in the epidural space and to observe the pattern of spread on anteroposterior and lateral views. Fluoroscopic image of the final level of contrast was recorded (Fig. 1). When the L4 and L5 endplates were not squared simultaneously in patients with distorted anatomy, we used L5 as the datum point. Contrast spread pattern evaluation included right or left side spread at the highest level of spread. After confirmation of epidural spread, a total volume of 5 ml of injectate containing 0.75% ropivacaine, dexamethasone, hyaluronidase 750 IU, and normal saline was infused. After the procedure, patients were observed for any adverse effects. For each patient, pain scores before the block and 30 minutes after the block were recorded using a numerical rating scale (NRS, ranging from 0 = no pain to 10 = absolutely intolerable pain). Epidurogram patterns of each patient were analyzed by experienced physicians who were not involved in this study.

### 2.3 Statistical analysis

All data were analyzed using SAS 9.4 (SAS Institute, Cary, NC). Data are expressed as mean ± standard deviation (SD) or number (proportion) as appropriate. Demographic data for the 3 groups were compared using Kruskal-Wallis rank sum test, Chi-square test, Fisher exact test or analysis of variance. The 3 different needle gauges, lesion severity, contrast spread level, and pain severity were analyzed using logistic regression analysis. A *P* value less than .05 was considered statistically significant.

## 3 Results

**Figure 2:**
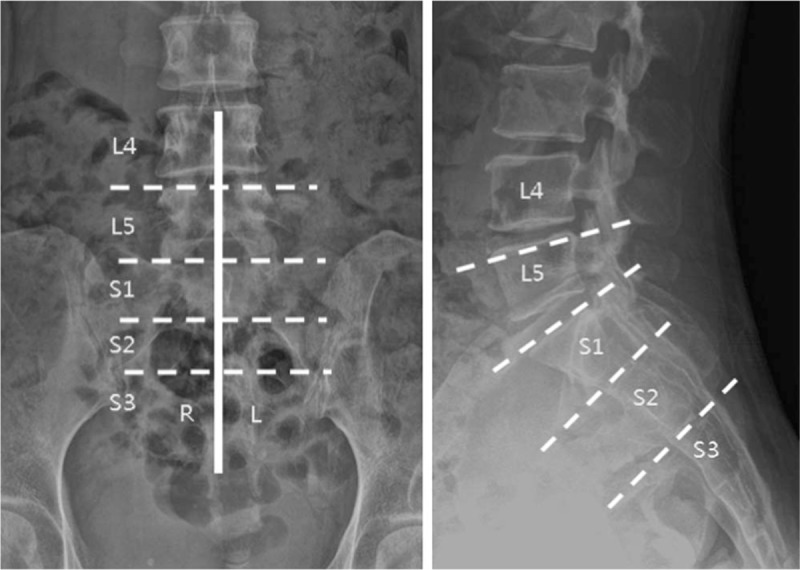
Schematic drawing for analysis of the level of epidural contrast spread. The dotted line indicates the range of epidural spread level. The bold line indicates the midline of the vertebral body. L4, includes the level above the upper margin of L5; L5, includes the level between the upper margin of L5 and the L5-S1 intervertebral space; S1, includes the level between the upper margin of S1 and the S1-2 intervertebral space; S2, includes the level between the upper margin of S2 and the S2-3 intervertebral space; S3, includes the level below the upper margin of S3. R indicates right side spread at the highest spread level; L indicates left side spread at the highest spread level.

Of the 80 patients assessed for eligibility, 7 were excluded because of poor fluoroscopic images (n = 5), failed caudal injection due to epidural venogram (n = 1), and anatomical variation in the sacral hiatus (n = 1). Thus, a total of 73 patients were finally analyzed. Patients were categorized into the following groups based on the needle gauge used for the caudal block: Group A, 23 gauge needle (n = 26); Group B, 20 gauge needle (n = 24); Group C, 17 gauge needle (n = 23) (Fig. 1). The demographic data of the patients are summarized in Table 1. Age, sex, height, body mass index, diagnosis, lesion level, lesion severity, and duration of pain did not differ among the 3 groups (Table 1). Clinical data are summarized in Table 2. Contrast spread pattern was analyzed using the following criteria: L4 - included the level above the upper margin of L5; L5 - included the level between the upper margin of L5 and the L5-S1 intervertebral space; S1 - included the upper margin of S1 and the S1-2 intervertebral space; S2 - included the level between the upper margin of S2 and the S2-3 intervertebral space; S3 - included the level below the upper margin of S3 (Fig. 2). All patients showed cephalic spread of contrast. Cephalic spread of contrast beyond L5 was seen in 26.9% patients in group A, 41.7% in group B, 39.1% in group C, and 35.6% overall; the differences among the groups were not statistically significant (Table 2). Contrast spread pattern to the right or left side was different between the groups (*P* = .017). Pain severity (NRS) before the block and 30 minutes after the block was not significantly different among the 3 groups (Table 2). None of the patients experienced dural puncture or neurologic sequelae.

**Table 1:**
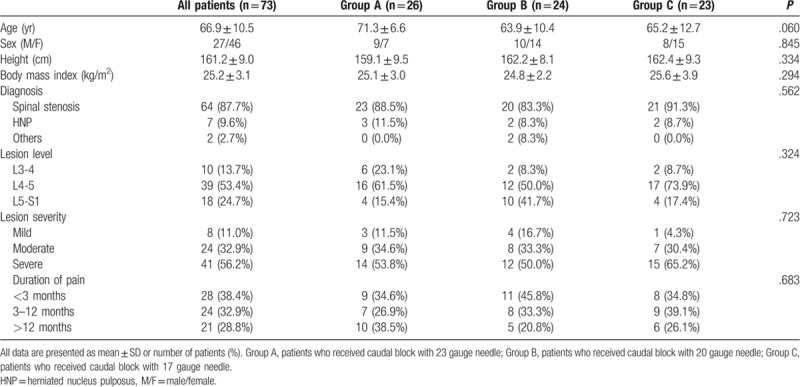
Patient demographics.

**Table 2:**
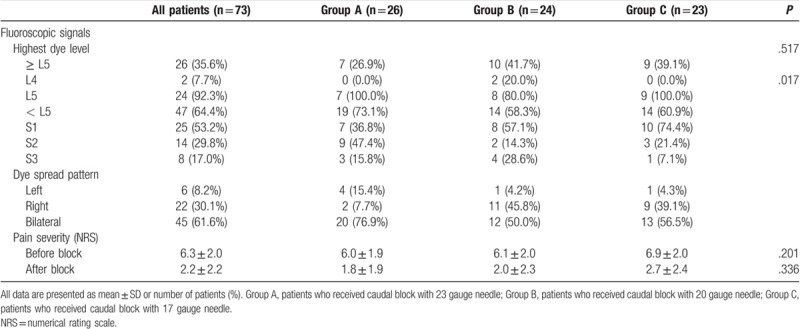
Clinical data of patients in the 3 groups.

## 4 Discussion

In this study, we investigated the influence of needle gauge on fluoroscopic epidural spread and assessed the correlation between the level of spread and analgesic efficacy in patients undergoing caudal block. No significant differences were observed among the 3 different needle gauges in terms of the highest level of epidural spread or analgesic efficacy among the 3 different needle gauges.

Epidural injection is known to reduce pain and improve functional status in patients with lower back and radicular pain due to spine pathology.^[2,13]^ Its efficacy depends on the precise delivery of the medication to the presumed site of the pathology. Among the several approaches for epidural injection, the caudal approach has been reported to have multiple advantages.^[14,15]^ It can reach the dorsal and the ventral epidural space^[14]^. Additionally, it is safe for managing post-surgery syndrome.^[2,16]^ The caudal approach is an easy way to deliver medications in case of multi-level spine pathology and in patients with severely narrowed lumbar canal stenosis. To facilitate delivery of the injectate to a distant target level, injection of large volumes or placement of epidural catheter through the caudal route have been evaluated.^[5,9,17]^ Kim et al^[17]^ observed that injecting progressively increasing volume (from 10 ml to 50 ml) with a 22 gauge needle showed no further cephalic spread with each subsequent injection, although it led to repainting of the path of the previous injections due to minimal resistance in the cephalic direction, anatomic variations, and Starling effect of the epidural space. In case of epidural catheter placement through the caudal route, other risks should be considered. The dural sac usually terminates between the S1 and S2 vertebrae. However, in 1% to 5% of patients, the dural sac terminates at S3 or below.^[18–20]^ Additionally, less than 5% of patients with low back pain or sciatica have perineural cysts that communicate with the dural sac, which is filled with cerebrospinal fluid.^[19,21]^ In these patients, there is the potential risk of inadvertent dural puncture or intrathecal injection. Thus, using an epidural catheter through the caudal route or deep insertion of the caudal needle for reducing the distance to the target lumbar pathologic site is not a completely safe method.

In this study, we tried to evaluate the effect of 3 different needle gauges on the level of epidural contrast spread. Since nerve root compression or irritation due to the spinal pathology mainly occurs above L5 or S1,^[22]^ we made the datum point of comparison of contrast spread as L5. We observed that high dye levels beyond L5 were seen in 26.9% of patients in group A, 41.7% of patients in group B, 39.1% of patients in group C, and 35.6% of all patients, with no significant difference among the needle gauge groups. Clinical effect of the caudal block was not related to lesion severity, level of epidural contrast spread, or the needle gauge. The injected contrast volume (3 ml) in this study was small compared with that in other caudal injection studies.^[5,14,17,23]^ This might have resulted in the lower proportion of patients with a high level of spread in this study. In the case of contrast levels below L5, the clinical effect of the caudal block might have been due to the greater cephalad spread after subsequent injection of medication. In a study that performed ultrasonography assessment of the injectate after caudal injection, secondary horizontal redistribution and longitudinal cranial spread were observed, probably caused by CSF rebound shift and epidural pressure change, which can lead to the difference in the initial and final block levels.^[14]^ Regardless of the direction of contrast spread among the 3 groups, the highest spread level and the analgesic efficacy were not significantly correlated with each other. It is not clear if the laterality of contrast spread is related to the needle gauge or other technical factors, as we did not record the direction of the needle tip at the insertion site. However, Jo et al reported that the caudal epidural flow pattern is not correlated with the laterality of the pain.^[24]^ Kwon et al reported that needle tip positioning to either the right or left side was not related to epidural drug spread pattern during caudal injection.^[25]^

This study had several limitations. First, we did not rule out the effect of the angulation of the lumbosacral junction on injectate spread. It can be an obstacle to cephalic spread. However, we did not measure the C-arm direction for deciding the spinal spread level. Second, we evaluated epidural spread with only 3 ml of contrast, as we felt that the subsequent 5 ml injectate might dilute the previous contrast distribution, and thus affect analysis of the spread levels. In addition, the contrast and injectate had different viscosities that might have caused bias regarding the range of spread. Third, the follow-up period after injection for evaluating the efficacy of the caudal block was short. Fourth, each patient consumed various analgesics or underwent interdisciplinary management that might have affected the severity of pain after the block. Fifth, the needle types were different and the influence of needle gauges higher than 23 or lower than 17 was not evaluated. Sixth, we did not measure the epidural injection pressure. In an in vitro study, it was seen that higher injection pressures were generated with higher gauge epidural catheters, and was associated with better epidural spread.^[26]^ However, the conditions were different from those of the human epidural space, which can be affected by the severity of the spinal pathology and by individual differences in injectate absorption and distribution in the epidural space.^[4,27]^

The influence of epidural needle gauge on epidural spread during a caudal block was investigated in this study. There was no significant difference in the highest level of epidural spread or analgesic efficacy among the 3 different needle gauges. Further randomized studies on a larger sample size using various needle types are needed to investigate the level of epidural spread after injection of contrast and injectate. Determination of analgesic efficacy and long-term follow-up also need to be carried out in future studies.

## Acknowledgments

The authors would like to thank the SMC biostatistics team for their statistical assistance and supervision.

## Author contributions

**Conceptualization:** Hue Jung Park, Jin Young Lee.

**Data curation:** Ji Hye Kwon, Min Seok Oh, Hyun Joo Jung, Min Kyoung Cho, Jin Young Lee.

**Formal analysis:** Jin Young Lee.

**Investigation:** Ji Hye Kwon, Jin Young Lee.

**Methodology:** Min Seok Oh, Jin Young Lee.

**Supervision:** Woo Seog Sim, Hue Jung Park, Jin Young Lee.

**Validation:** Hyun Joo Jung, Jin Young Lee.

**Visualization:** Min Kyoung Cho, Jin Young Lee.

**Writing – original draft:** Jin Young Lee.

**Writing – review & editing:** Hue Jung Park, Jin Young Lee.

Jin Young Lee orcid: 0000-0003-1499-2197.
